# The relationship between physical activity and interpersonal distress in college students: the chain mediating role of self-control and mobile phone addiction

**DOI:** 10.1186/s41155-023-00261-3

**Published:** 2023-08-02

**Authors:** Chong Liu, Zongchen Sun

**Affiliations:** College of Physical Education, Yangzhou University, Yangzhou City, Jiangsu Province 225000 People’s Republic of China

**Keywords:** College students, Physical activity, Interpersonal relationships, Self-control, Mobile phone addiction

## Abstract

**Objective of the study:**

Interpersonal relationships, as an important variable affecting the physical and mental health and future development of individuals, were used to construct a structural equation model between physical activity and interpersonal relationships in order to help college students better adapt to society and achieve a high level of mental health.

**Methods:**

SPSS 27.0 software was used to statistically analyze the data, and Amos 28.0 software was used to construct the model between variables. The results showed that physical activity directly predicted the interpersonal relationship status of college students (β =  − 0.108, 95% CI [− 0.210, − 0.005]), and the chain mediating effect of physical activity → self-control → mobile phone addiction tendency → interpersonal relationship distress was significant (β =  − 0.012, 95% CI [− 0.033, − 0.003]). The results of this study suggest that physical activity may be viewed as an effective intervention strategy to mitigate the interpersonal challenges that college students may face in the future.

## Introduction

Interpersonal relationship is a kind of psychological relationship formed between people through interaction and interaction, reflecting the psychological state of individuals or groups seeking to satisfy their needs (Zhang, 2007), and research has found that interpersonal relationship is one of the key factors affecting college students’ psychological health (Zhu et al., 2013). A large amount of evidence shows that good interpersonal relationships are positively related to positive factors such as subjective well-being (Zhang et al., 2021) and academic achievement (Fan, 2012), while interpersonal relationship distress is positively related to negative factors such as loneliness (Kim et al., 2017) and mobile phone addiction (Liao et al., 2016). In China, if an adolescent has a high school education or higher, he or she will have 12–17 years of schooling, and the college level is an important time for these adolescents to make the transition to society. According to the definition of health by the World Health Organization, health includes not only physical and mental health but also good social adjustment, and the quality of interpersonal relationships, as an important indicator of good social adjustment, will directly affect the physical and mental health and social adjustment ability of individuals. Therefore, solving the troubling problems in interpersonal relationships is of great significance to the social development of college students, and promoting the healthy development of interpersonal relationships among college students is not only beneficial to the physical and mental health of individuals, but also helps the college student group to adapt to the social environment more smoothly in the process of socialization.

Therefore, the establishment of healthy interpersonal relationships plays an extremely important role in the psychological health and social adaptability of college students. This paper explores the influence of physical activity on interpersonal relationships of college students from the perspective of sports, aiming to help people better understand the relationship between the two and to provide theoretical guidance for physical activity to intervene in the problem of interpersonal relationship distress among college students.

## Physical activity and interpersonal relationships

According to Maslow’s hierarchy of needs theory, when a person satisfies the low-level needs of physiology and security, he or she will establish emotional ties with others in order to further gain a sense of love and belonging. Compared with college students with interpersonal problems, those who have good interpersonal relationships can establish deep emotional ties with others more easily; therefore, it may be relatively difficult for those college students who are troubled in interpersonal relationships to satisfy their social needs. Physical activity has been increasingly recognized by the general public as a healthy lifestyle, and to improve health and reduce the risk of chronic disease and depression, the World Health Organization recommends at least 150 min of moderate-intensity aerobic exercise per week for people aged 18–64 (WHO, 2010). Physical activity conducted in accordance with scientific principles as a means to improve people's physical and mental health has become a consensus (Biddle et al., 2019; Tomporowski & Pesce, 2019), and Magulandam (2019) confirmed that physical activity can promote interpersonal relationships and reduce negative emotions through an 18-week physical dance intervention, and Zheng et al. (2022) similarly confirmed that physical activity can improve interpersonal relationships among college students through a 12-week basketball exercise intervention. Based on the above analysis, this study proposes H1: physical activity can negatively predict interpersonal relationship distress among college students.

## Physical activity, self-control, and interpersonal relationships

Self-control refers to the ability to change one's reactions, especially the ability to make one’s reactions conform to ideals, values, morals, and other standards and to support the long-term pursuit of goals (Baumeister et al., 2007). It is the process by which individuals overcome their own desires and needs and change their inherent behaviors and ways of thinking, the process by which one behavior and way of thinking replaces another. Cross-sectional studies show a positive relationship between physical activity and self-control (Schöndube et al., 2017; Zhu et al., 2014), and behavioral and physiological evidence confirms that physical activity enhances inhibition, which is a core component of self-control (Tao, 2019). Research has shown that people with high self-control get along better with others and have better interpersonal relationships (Murphy and Eisenberget, 1997; Tangney et al., 2004). In addition, self-control helps individuals to remain calm during conflicts and disagreements, to solve problems rationally, and to avoid impulsive relationships. Recent studies have also confirmed that physical activity can improve college students' interpersonal relationships directly by increasing self-control, and indirectly through self-control (Magulandam et al., 2019). Based on the above analysis, this study proposes that H2: self-control can play a mediating role between physical activity and interpersonal relationship distress among college students.

## Physical activity, mobile phone addiction, and interpersonal relationships

According to the 50th Statistical Report on the Development of the Internet in China released by the China Internet Information Center, as of June 2022, the number of Chinese Internet users reached 1.051 billion, of which 1.047 billion were mobile phone users, accounting for 99.6% of the total number of Internet users(CNNIC, 2022). As a digital product that facilitates people's life intelligently, mobile phone was originally invented to facilitate people's life and can make people break through the limitation of distance to communicate, but with the development of technology, mobile phone may also bring some negative effects—"mobile phone addiction”. While addiction is a behavioral process that cannot be repeatedly controlled and continues despite significant negative consequences (Goodman, 1990), mobile phone addiction is a behavior in which one becomes addicted to the use of mobile phones at the expense of other aspects of life (Goswami & Singh, 2016), making its function go against its original invention in this behavioral process.

A recent meta-analysis confirmed a negative association between physical activity and mobile phone addiction among young people, regardless of the time of data collection, country or region, or population (Xiao et al., 2022). Studies from different regions of China have found that physical activity reduces mobile phone addiction among college students, and moderate intensity physical activity significantly improves this phenomenon (Li et al., 2023; Yang et al., 2020). Numerous studies have found that there is a significant positive correlation between mobile phone addiction and interpersonal relationship distress (Liao et al., 2016; Tang et al., 2018) and a negative correlation between healthy interpersonal relationship (Kim et al., 2017), and physical activity can directly improve college students’ mobile phone addiction behavior, and mobile phone addiction behavior can influence individuals’ interpersonal relationship status. Based on the above analysis, this study proposes that H3: the tendency of mobile phone addiction can play a mediating role in the process of college students' physical activity affecting interpersonal relationships.

## Current study

**Fig. 1:**
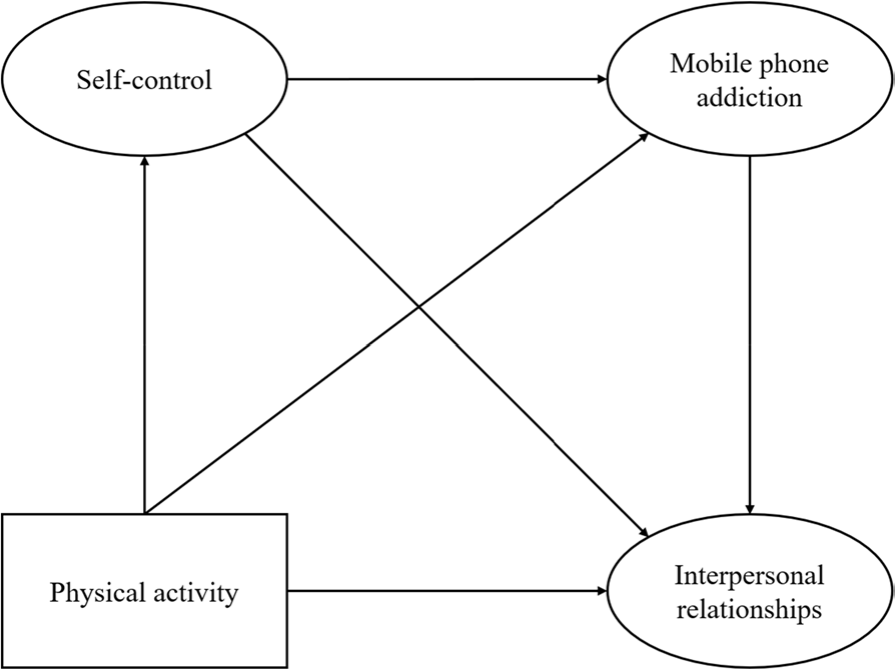
Hypothetical model

Most of the existing studies have examined interpersonal relationships as a negative effect of mobile phone addiction (Chen et al., 2016; Przybylski & Weinstein, 2013), and there are more articles that use mobile phone addiction as a simple mediating variable to explore the mechanisms between the two, but there are fewer articles that use self-control as a mediating variable to explore the potential pathways by which physical activity affects interpersonal relationships. There is abundant evidence that addictive behaviors are strongly related to self-control (Kim et al., 2008; Song & Park, 2019), and existing studies suggest that self-control negatively predicts mobile phone addiction (Kim & Sohn, 2014; Van et al. 2015). However, no literature has been reported to explore the linkage mechanism between self-control and mobile phone addiction in the process of interpersonal relationships among college students. In summary, based on the above analysis, this study proposes that H4: college students’ tendency to self-control and mobile phone addiction play a chain mediating role between physical activity and interpersonal relationship status. The hypothetical model Fig. 1 is as follows:

## Method

### Participants

A convenience sampling method was used to release an electronic questionnaire to 528 college students in a university in Jiangsu Province. Before releasing the electronic questionnaire, each participant signed an informed consent form, and the recovered questionnaires were screened according to the inclusion and exclusion criteria. 488 valid questionnaires were acquired in the study, with an effective rate of 92% (370 males (age 19.15 ± 1.15) and 118 females (age 19.41 ± 1.42), mean age 19.21 ± 1.22 years).

### Instruments

#### Demographics questionnaire

A self-administered demographic questionnaire was used to investigate the gender, age, and grade level information of the subjects.

#### Physical activity rating scale

The physical activity level scale, prepared by Japanese scholar Koyo Hashimoto and translated and revised by Deqing Liang, was used, with a retest reliability of 0.82 (Yin & Wen, 2021). A total of 3 entries in a single dimension were exercise intensity, exercise time, and exercise frequency. In order to describe the physical activity level of college students more carefully, the 5-point Likert scale was scored, and the formula was: physical activity amount = exercise intensity × (exercise time − 1) × exercise frequency, and the higher the score, the greater the physical activity amount. The Cronbach's α coefficient of this scale in this study was 0.634.

#### Self-control scale

The Self-Control Scale for College Students revised by Shuhua Tan and Yongyu Guo (2008) was used, which has 19 entries and consists of 5 dimensions: impulse control, healthy habits, resisting temptation, focusing on work and study, and abstaining from recreation. A 5-point Likert scale is scored, and a higher total score indicates greater self-control. The Cronbach's alpha coefficient for this scale in this study was 0.841.

#### Comprehensive interpersonal relationship diagnostic scale

A comprehensive interpersonal relationship diagnostic scale (Yan et al., 2022) developed by Zheng was used, which has 28 entries and consists of four dimensions: interpersonal conversation, interpersonal friendship, treating people well, and heterosexual interaction. The higher the total score, the more serious the interpersonal distress problem. The Cronbach's alpha coefficient of the scale in this study was 0.925.

#### Mobile phone addiction tendency scale

The Mobile Phone Addiction Tendency Scale for College Students developed by Xiong et al. (2012) was used. The scale consists of 15 items, which can be divided into four dimensions: withdrawal symptoms, emergent behavior, social soothing, and mood change. The higher the score, the greater the individual's tendency to become addicted to mobile phones. The Cronbach's alpha coefficient of the scale in this study was 0.909.

#### Statistical analysis plan

SPSS 27.0 and AMOS 28.0 from IBM were used for statistical analysis of the data. The normality of the variables was examined using the Shapiro–Wilk test, and gender differences between men and women were compared using an independent samples t-test if the data were normal, and Mann–Whitney *U* if the data were not normal, and then Pearson correlation analysis was used to examine the relationship among college students' physical activity, self-control, interpersonal relationship, and mobile phone addiction tendency, and then the structural equation model was constructed by Amos software for path analysis of the hypothesized model, and finally the Bootstrap method was used to The chain mediating role of self-control and mobile phone addiction tendency in physical activity and interpersonal relationship among college students was tested by Bootstrap method, and *P* < 0.05 was set as the statistical result with significance.

## Results

### Control and testing of common method deviations

Since all data in this study were reported by the subjects themselves, the data needed to be controlled and examined for common method bias. Some of the questions in the distributed questionnaire were reverse scored and irrelevant questions were set to control for the effect of common method bias in the data collection process. The measured experimental data were incorporated into SPSS 27.0 and tested for common method bias using Harman’s one-way test, and the results showed that there were 13 factors with characteristic roots > 1, and the largest factor had an explained variance of 17.76% (< 40%), and we calculated the CR and AVE values for each scale to give evidence of convergent and discriminant validity of the scales. The CR = 0.77 and AVE = 0.41 for Self-Control Scale, CR = 0.87 and AVE = 0.64 for Mobile Phone Addiction Tendency Scaled, Comprehensive Interpersonal Relationship Diagnostic Scale had a CR = 0.90 and AVE = 0.69,which inferred that the data in this study did not have serious common method bias.

### Normality test and differences between groups

The data were imported into SPSS and each variable was tested for normality using the Shapiro–Wilk test, and the results suggested that the *p* values for the normality test were < 0.001, meaning that the data did not meet the normality requirements, and Mann–Whitney *U* was applied to compare the differences between groups for each variable (Table 1). The data showed that the gender differences in self-control and interpersonal relationships among college students were not significant (*p* > 0.05), while the gender differences in physical activity and cell phone addiction tendency among college students were significant (*p* < 0.01). The Wilcoxon rank sum test showed that the rank mean for physical exercise was higher for males than for females (male = 260.84, female = 193.28), and the rank mean for the tendency of mobile phone addiction was lower than for females (male = 234.77, female = 275.00).

**Table 1:** Gender differences between variables

Variables	Gender			
	PA	SC	IR	MPAT
Mann–Whitney *U*	15785.50	20321.50	19364.00	18231.00
Wilcoxon *W*	22806.50	27342.50	87999.00	86866.00
*Z*	− 4.55	− 1.13	− 1.86	− 2.71
Asymptotic saliency (two-tailed)	0.00	0.26	0.06	0.01
Male	12(6,32)	57(54,64)	5(0,12)	45(36,49)
Female	6(3,18)	57(53,62)	8(3,11)	48(40,52)

*PA* Physical activity rating scale score, *SC* Self-control scale score, *IR* Comprehensive interpersonal relationship diagnostic scale score, *MPAT* Mobile Phone addiction tendency scale

### Correlation analysis between the variables

The results of Pearson correlation analysis (Table 2) suggested that physical activity was positively correlated with self-control (*r* = 0.20, *p* < 0.01) negatively correlated with interpersonal distress (*r* =  − 0.17, *p* < 0.01) and the propensity for mobile phone addiction (*r* =  − 0.21, *p* < 0.01), which is consistent with the previous theoretical analysis. The remaining variables were two-by-two correlated with each other, allowing for the next step of mediating effects analysis (Table 2).

**Table 2:** Descriptive statistics and correlation coefficients of the total score of each study variable

Variables	PA	SC	IR	MPAT
PA	–			
SC	0.20**	–		
IR	− 0.17**	− 0.28**	–	
MPAT	− 0.21**	− 0.32**	0.24**	–

*P < 0.05; **P < 0.01; ***P < 0.001; *PA* = Physical Activity Rating Scale score, *SC* Self-control scale score, *IR* Comprehensive interpersonal relationship diagnostic scale score, *MPAT* Mobile phone addiction tendency scale

### A test of mediating effects of self-control and interpersonal distress on mobile phone addiction

**Fig. 2:**
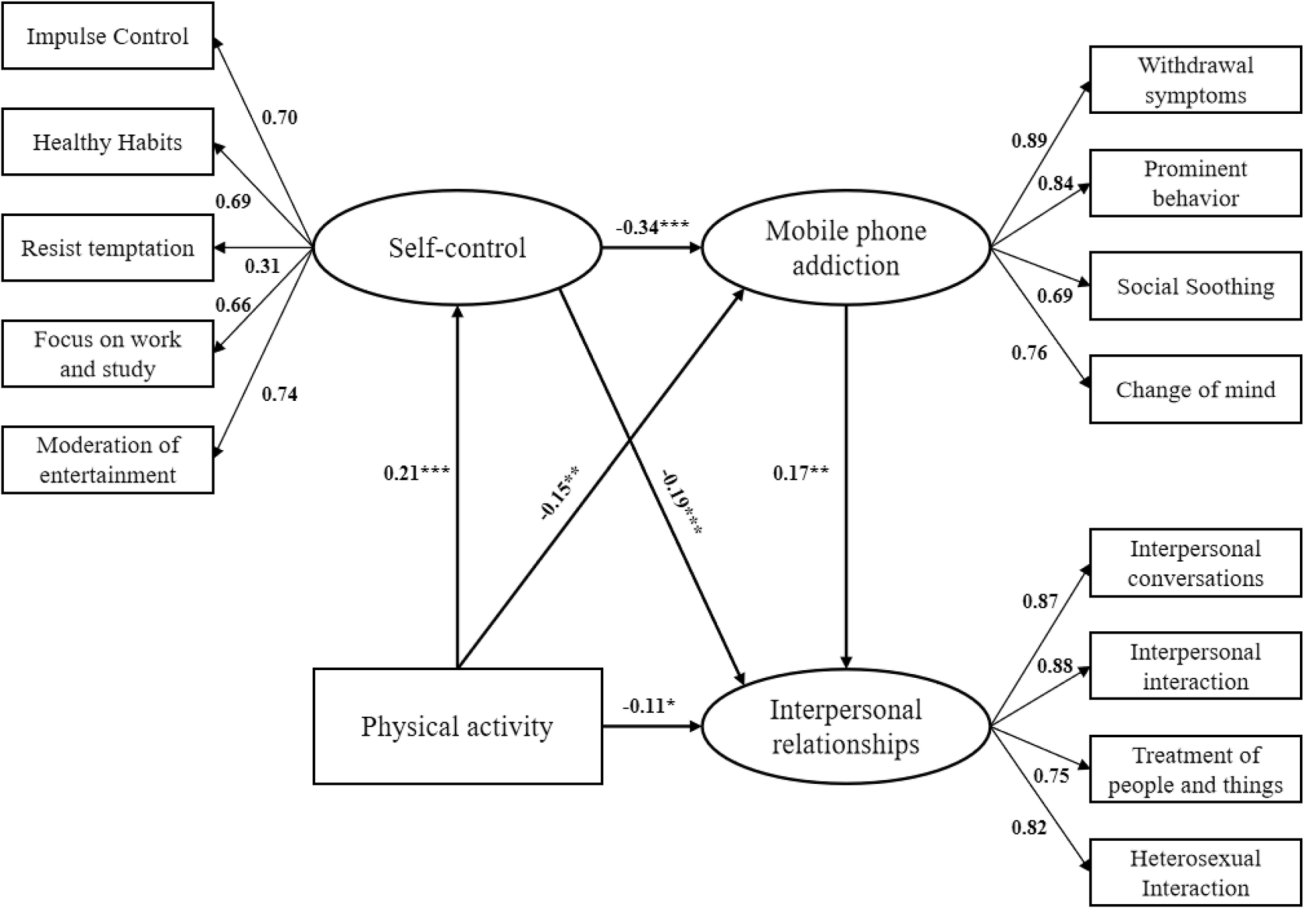
Effect diagram of chain intermediary model

The model was developed as shown in Fig. 2, and the overall model fit index was χ ^2^/df = 3.945, CFI = 0.931, TLI = 0.913, and RMSEA = 0.078, indicators suggesting that the model fit the data well. Self-control and interpersonal distress were used as mediating variables, and physical activity and mobile phone addiction were used as independent and dependent variables, respectively, and the mediation effects of self-control and interpersonal distress were tested at 95% confidence intervals using the nonparametric bias-corrected percentile Bootstrap method with 5000 samples. The results (Table 3) suggest that the total effect of physical activity on interpersonal distress was significant (β =  − 0.185, *p* < 0.01) and the direct effect of physical activity on interpersonal distress among college students was significant, i.e., physical activity was a direct predictor of interpersonal distress (β =  − 0.108, *p* < 0.01), thus validating H1. All remaining path coefficients reached the significant level (Table 3) in the model (Fig. 2) in which self-control and mobile phone addiction tendency play a mediating role in physical activity and interpersonal relationship distress among college students, this mediation role has three paths: Path 1: separate mediation of self-control, the results suggest that self-control plays a partially mediating role in the process of physical activity influencing mobile phone addiction ab =  − 0.040, 95% CI [− 0.099, − 0.008], validating H2; Path 2: Separate mediating role of mobile phone addiction tendency, the results suggest that college students' mobile phone addiction tendency partially mediates the process of physical activity influencing interpersonal distress ab =  − 0.025, 95% CI [− 0.068, − 0.002], validating H3; Path 3: Chain mediating role of self-control → mobile phone addiction tendency, the results suggest that self-control → mobile phone addiction tendency to play a chain mediating role in the process of physical activity affecting interpersonal relationship distress ab =  − 0.012, 95% CI [− 0.033, − 0.003], validating H4. The above results suggest that self-control and mobile phone addiction tendency have an indirect influence in the process of physical activity alleviating interpersonal relationship distress among college students. In addition, self-control and mobile phone addiction tendency further influenced the effect of physical activity on alleviating interpersonal distress through a chain-mediated mechanism.

**Table 3:** Path and effect values of the intermediary model

Paths	Effect	Boot SE	Boot LL CI	Boot UL CI	Effectiveness Ratio
Direct effect	− 0.108	0.052	− 0.210	− 0.005	58%
Indirect effects1	− 0.040	0.023	− 0.099	− 0.008	22%
Indirect effects2	− 0.025	0.016	− 0.068	− 0.002	14%
Indirect effects3	− 0.012	0.007	− 0.033	− 0.003	6%
Total indirect effect	− 0.076	0.023	− 0.132	− 0.040	42%
Total effect	− 0.185	0.049	− 0.276	− 0.084	–

## Discussion

### Gender differences in physical activity and mobile phone addiction among college students

The purpose of this study is to explore the mechanisms underlying the influence of physical activity on interpersonal relationship distress in a college student population. The data analysis in this paper found significant gender differences in scores for physical activity and mobile phone addiction tendencies, with male college students being higher than female college students in terms of amount of physical activity and lower than female college students in terms of mobile phone addiction tendencies. Thomas (1988) argues that gender differences in physical activity are due to hormonal differences. And the combination of environment causes that men's physiological structure is generally stronger than women’s, and men’s better physical quality is more suitable for activities that require strength and size. In addition, the attitude of physical activity and sports venues are also one of the reasons for the gender difference in physical activity among college students, research shows that male college students have more positive attitudes toward physical activity than female college students, and the more positive attitudes toward physical activity, the more frequently they exercise. In terms of sports venues, at present, the sports venues in Chinese colleges and universities are concentrated in high-intensity and confrontational sports such as basketball and soccer, while girls tend to prefer quiet sports such as yoga and dance, but there is a lack of such venues, which leads to insufficient external motivation for female college students to participate in sports, and all these factors will restrict the exercise behavior of female college students, and thus lead to gender differences in physical activity among college students. This result is also consistent with previous studies (Liu, 2021).

The findings also suggest that female college students have significantly higher scores of mobile phone addiction than male college students, which is consistent with previous studies (Sekhon, 2018). One study suggests that women use mobile phones more frequently than men because they rely on them more than men to establish and maintain social relationships and express their emotions (Pawłowska & Potembska, 2011), and some scholars suggest that women are more likely to be addicted to mobile phones than men because they have less self-control than men (Davey et al., 2020), but the self-control scores in this paper do not support this idea.

### Physical activity promotes interpersonal relationships

Sport, as a uniquely human social activity, is by its very nature a socio-cultural activity and a form of interpersonal interaction. Physical activity helps to channel our mental state into a positive direction, including reducing stress, relieving tension, and dispelling depression, among many other benefits. According to the theory of teamwork, participation in group sports activities can further strengthen the cohesiveness between students and friends through conscious communication. In the process of such interaction, mutual understanding and trust gradually increase, which helps to deepen friendship and emotional bonds during and after physical activity, thus establishing healthy, harmonious and stable interpersonal relationships and improving interpersonal skills. In addition, the theory of interpersonal attraction is also an important factor. Regular participation in physical activity can improve the physical health and image of individuals, which makes them more attractive in their interactions with others and thus promotes the development of interpersonal relationships (Tolman, 2016).

### Separate mediating roles for each of self-control and mobile phone addiction

In this study, correlational analyses and mediated effects tests yielded three pathways by which physical activity influences interpersonal distress, with self-control as a separate mediating variable in the first pathway. This is a less extensively studied pathway, and the effect analysis of the mediation model suggests that self-control partially mediates the effect of physical activity and interpersonal distress, and this mediation accounts for 14% of the total effect of physical activity on interpersonal distress in this model and 33% of the total indirect effect. According to Baumeister’s power model theory of self-control, self-control is a limited resource, and individuals need to improve self-control through continuous practice in daily life, and psychology can improve the mental health and well-being of many people through the pathway of self-control based on its theoretical model (Baumeister, 2007). Physical activity is an effective form of self-control training, and through regular exercise, individuals can learn to better manage their behavior and emotions and thus behave more maturely and consistently in interpersonal interactions. The results suggest that we can start with physical activity for college students when facing the problem of interpersonal distress among college students. Provide college students with more diverse forms of sports and sports venues, create a good sports atmosphere, and allow students to help each other in sports and develop a spirit of cooperation. Improve their interpersonal relationship by improving their self-control ability while enhancing their physical fitness.

The second path was to use the propensity to mobile phone addiction as a separate mediating variable. In the results of this study, the mediating effect test revealed that the propensity to mobile phone addiction played a partially mediating role in physical activity and interpersonal relationship distress, and this mediation accounted for 22% of the total effect of physical activity affecting interpersonal relationship distress and 52% of the total indirect effect in this model. Suffering from mobile phone addiction is likely to trigger some negative psychological effects, such as loneliness and anxiety. The existing literature review and meta-analysis confirmed that students with mobile phone addiction are more likely to have negative emotions such as anxiety, depression, and impulsivity among the college population (Li et al., 2020), which is consistent with previous studies (Smetaniuk, 2014). The ability of physical activity to positively promote interpersonal relationships (Kim et al., 2017; Magulandam et al., 2019) and improve mobile phone addiction (Li et al., 2023; Yang et al., 2020) among college students has been supported by numerous research evidences, according to self-identity theory, when individuals reduce their mobile phone addiction through physical activity, they will have the opportunity to engage in more social activities, expand their networks, and make more meaningful connections in real life. By joining sports clubs and participating in group sports programs, individuals will find friends who share their values and strengthen their sense of self-identity, thus enhancing their self-esteem and psychological well-being. This finding suggests that when facing interpersonal relationship problems among college students, schools, families, and society can try to start with the problem of mobile phone addiction.

The college student group is in the critical period of socialization process, therefore, how to improve their interpersonal relationship problems is of great significance to their development. On the one hand, physical activity can effectively reduce college students’ tendency of mobile phone addiction and thus indirectly improve their interpersonal relationships. On the other hand, physical activity can improve the interpersonal skills of college students, so that they can better adapt to the social environment and promote overall development. The improvement of self-control and the reduction of mobile phone addiction tendency together provide strong support for college students to achieve all-round health.

### The chain mediating role of self-control and mobile phone addiction

The third pathway, which is also the main finding of this study, is the chain mediating role of self-control and the propensity for mobile phone addiction in the process of physical activity improving interpersonal distress among college students. Many previous studies have found self-control to be an important mediating variable in improving mobile phone addiction (Kim et al., 2008; Song & Park, 2019). According to Maslow’s hierarchy of needs theory, when individuals have satisfied their security needs, they will pursue higher-level needs, such as love, friendship and belonging. College students with good interpersonal relationships can further pursue social and emotional needs on the basis of satisfying security needs. On the contrary, college students facing interpersonal relationship troubles tend to stay at the level of security needs and are more likely to rely on mobile phones to express their emotions and maintain social connections. Such dependence may lead to further deterioration of the problem and develop into mobile phone addiction. Therefore, it is important to pay attention to college students’ interpersonal relationships and their need levels to understand their behavior patterns. According to the limited resource model of self-control, direct evidence suggests that low self-control is detrimental to having and maintaining close interpersonal relationships, and high self-control leads to successful interpersonal relationships (Vohs, 2006). Overall, physical activity has a positive effect in promoting self-control and interpersonal relationships among college students. Improving self-control can effectively improve college students’ tendency of mobile phone addiction, which in turn alleviates interpersonal relationship distress. Meanwhile, the improvement of interpersonal relationships also helps to further reduce the phenomenon of mobile phone addiction. Therefore, the chain mediation model of self-control ability → interpersonal relationship distress proposed in this study has some feasibility. This finding has important insight and reference value for future exploration of interpersonal relationship problems of college students and their improvement measures.

## Limitations

This study explored the mechanisms underlying physical activity to improve interpersonal relationships in a young population through a cross-sectional survey, and although a structural equation model was constructed, the sample group still had limitations. Future research will need to further expand the sample size and validate the results of this study in older and adolescent populations. At the same time, this study also has another limitation. The research on interpersonal relationships in this study mainly focuses on the opposite sex, and the interpersonal interaction between homosexual or non-cis genders is lacking in this study. Future research needs to explore the interaction of interpersonal relationships within these groups in order to provide a more comprehensive understanding of this relationship for all groups.It is also noted that further insight is needed on the issue of interpersonal relationships due to cultural differences between China and the West. Still, this study provides a new way of thinking to improve the problem of interpersonal relationship distress among college students, and the findings of the model can be applied to a variety of practical scenarios, such as education, health, and socialization. By promoting physical exercise and self-control and reducing cell phone addiction, the quality and stability of interpersonal relationships can be enhanced, which can positively affect individuals' well-being and quality of life. It also provides a new perspective and theoretical framework for studying the mechanisms of interpersonal relationship promotion, and most importantly, this framework can be practiced in real-life interventions, which is of great significance for research in this field.

## Conclusion

Physical activity may directly improve interpersonal relationship distress problems among college students, or it may achieve the same goal through a chain-mediated effect of improving self-control and reducing the propensity for mobile phone addiction among college students. The results of this study suggest that physical activity can be viewed as an effective intervention strategy to alleviate the interpersonal challenges that college students may face in the future. Based on this research finding, physical education brings new perspectives and insights to address interpersonal relationship problems among college students.

## Abbreviations

PA: Physical Activity Rating Scale
SC: Self-Control Scale
IR: Comprehensive Interpersonal Relationship Diagnostic Scale
MPAT: Mobile Phone Addiction Tendency Scale
