# Correction: The relationship between physical activity and interpersonal distress in college students: the chain mediating role of self-control and mobile phone addiction

**DOI:** 10.1186/s41155-023-00270-2

**Published:** 2023-09-15

**Authors:** Chong Liu, Zongchen Sun

**Affiliations:** College of Physical Education, Yangzhou University, Jiangsu Province, Yangzhou City, 225000 People’s Republic of China

**Correction:** **Psicol. Refl. Crít. 36, 18 (2023)**

**https://doi.org/10.1186/s41155-023-00261-3**

Following publication of the original article (Liu & Sun, 2023), the authors reported an error in the Fig. 2, which have been corrected from:

Figure 2 presented a typing error in beta coefficient regression in the arrow Physical activity to Interpersonal relationships. Where it appears: 0.13** should figure -0.11* (the rounding of 0.108*). See the Fig. 2 corrected.

**Fig. 2:**
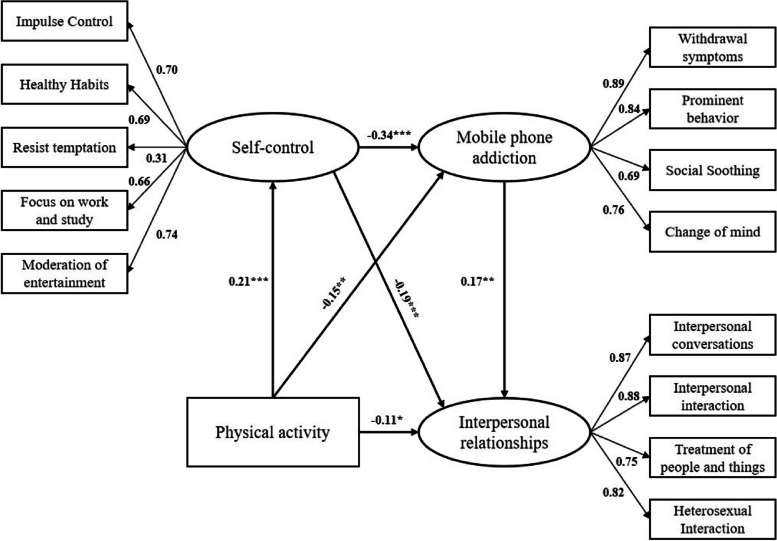
Effect diagram of chain intermediary model

The original article (Liu & Sun, 2023) has been corrected.
